# Coexistent frontal fibrosing alopecia with ophiasis pattern alopecia areata in a young female: A case report and review of the literature

**DOI:** 10.1002/ccr3.9165

**Published:** 2024-07-07

**Authors:** Mahsa Babaei, Ghasem Rahmatpour Rokni, Fatemeh Montazer, Nasim Gholizadeh

**Affiliations:** ^1^ Department of Medical Sciences Stanford University Stanford California USA; ^2^ Department of Dermatology Mazandaran University of Medical Sciences Sari Iran; ^3^ Department of Pathology Iran University of Medical Sciences Tehran Iran

**Keywords:** dermatopathology, frontal fibrosing alopecia, ophiasis pattern, topical corticosteroids

## Abstract

This study highlights the possibility of coexistent Frontal Fibrosing Alopecia and ophiasis pattern Alopecia Areata especially in young females which is a rare manifestation. A concomitant shared pathophysiology is suspected to underlie this association.

## Graphical abstract

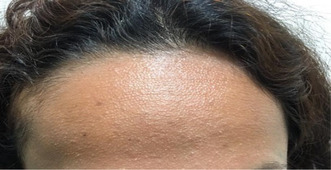

## 1 INTRODUCTION

Frontal fibrosing alopecia (FFA) is defined as band‐like cicatricial alopecia on the frontotemporal area of the scalp. The condition can be associated with perifollicular inflammation and alopecia of other areas, including the eyebrow, axillae, pubic region, and extremities. The disorder is histopathologically characterized by lymphocytic infiltrate around the isthmus and infundibulum with a decrease in the number of follicles, which are replaced by fibrosis.^1^

Although the exact underlying mechanism is not known, genetic and environmental factors are considered to be involved in FFA.^2^ The disorder mainly affects postmenopausal women and rarely affects younger females, males, or children.^3^ A final diagnosis is confirmed through clinical examination and histopathological studies.^4^ Proposed treatment options include corticosteroids, 5‐alpha‐reductase inhibitors, and hair transplantation.^5^, ^6^, ^7^

Alopecia areata (AA) is an autoimmune disease that destroys hair follicles. Ophiasis is a subtype of AA which manifests as a symmetric, band‐like pattern of hair loss on the occipital, temporal, and parietal areas of the scalp. This subtype most commonly occurs above the ears on the lateral area of the scalp and is refractory to conventional treatment with corticosteroids, immunotherapy, and minoxidil.^8^, ^9^ Studies have rarely reported coexistent FFA and AA in one case. In this paper, we report on a case of FFA associated with ophiasis.

## 2 CASE HISTORY/EXAMINATION

**FIGURE 1:**
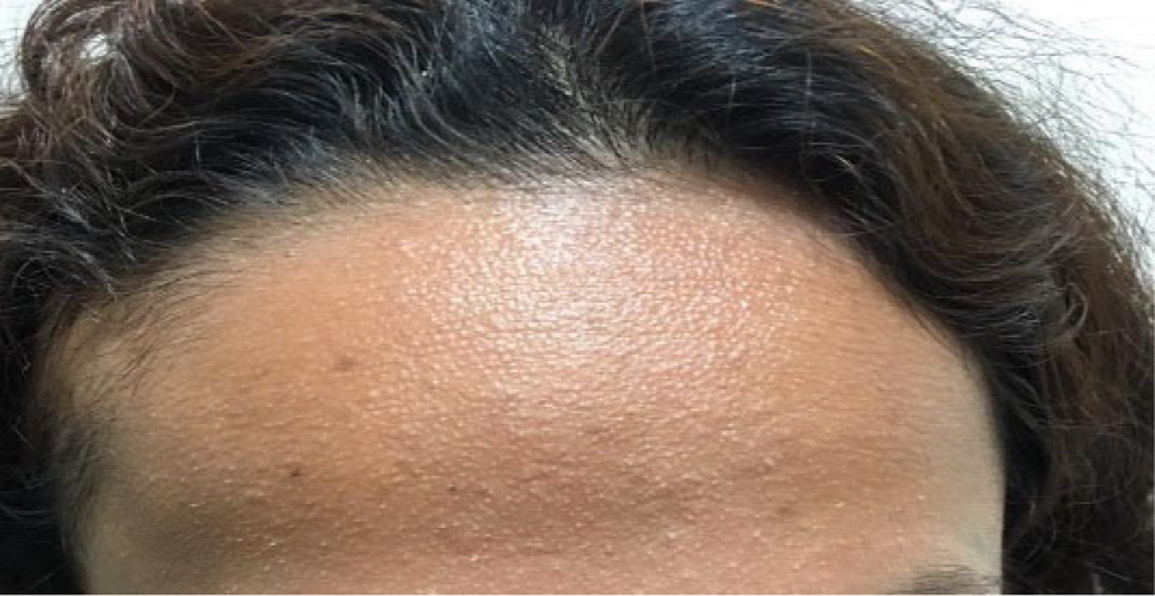
Skin‐colored papules at the front of the scalp and forehead.

**FIGURE 2:**
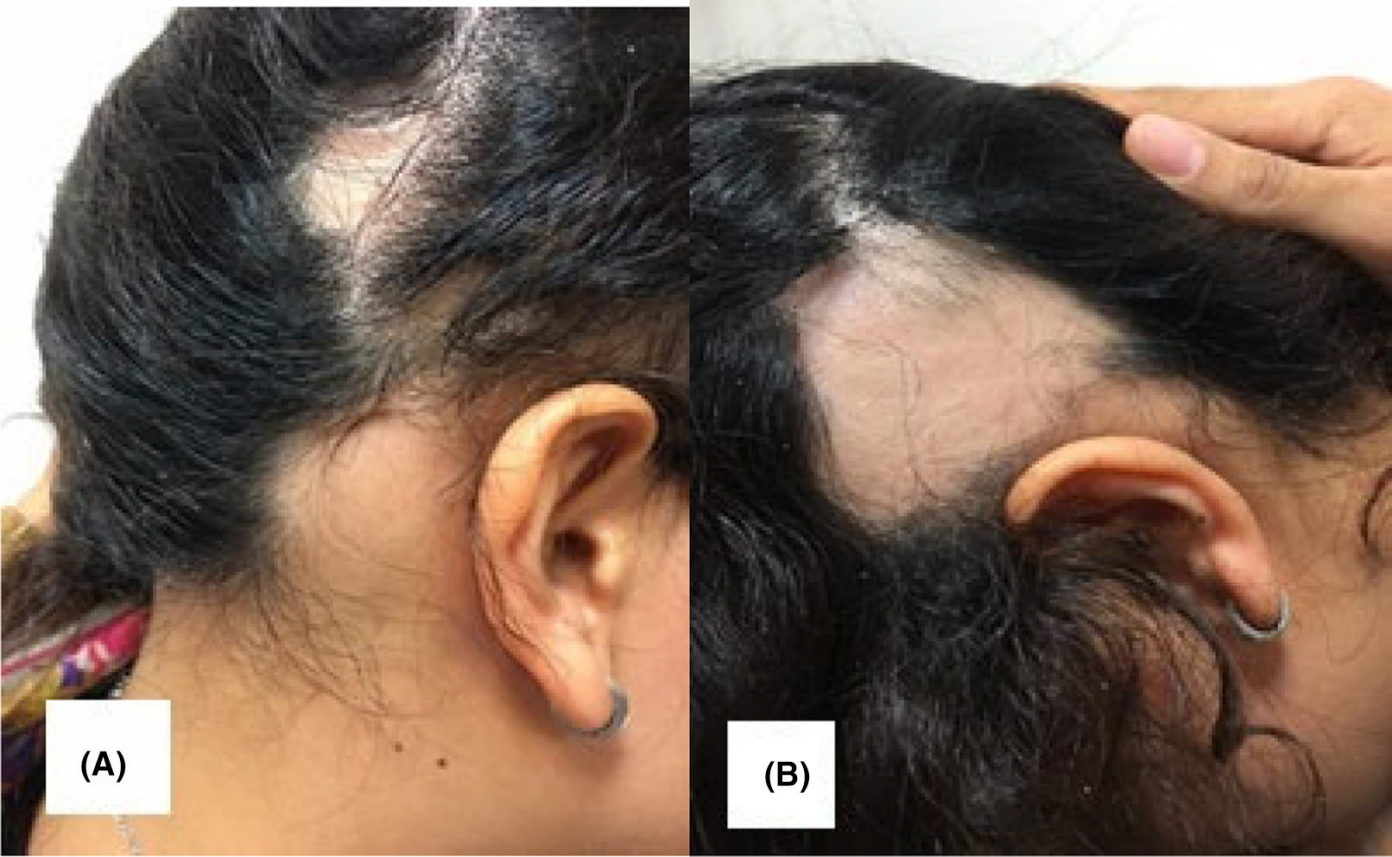
Patches of hair loss on the scalp.

An 18‐year‐old female presented to our clinic with a 2‐year history of development of skin‐colored papules on the scalp and the forehead (Figures 1 and 2). The lesions were otherwise asymptomatic and had not increased in size during this period. She has experienced several episodes of exacerbation that were triggered by emotional stress and the wearing of a head band. The patient also complained of patches of hair loss on the scalp commencing at 4 years of age in a waxing and waning pattern. Past medical history was unremarkable for any specific disorder and she reported no associated signs and/or symptoms. She had not previously sought medical treatment for these problems.

Clinical examination indicated three patches of hair loss, two in the temporal area bilaterally (6 × 6 cm), and one in the occipital area (2 × 3 cm) which featured an ophiasis pattern of alopecia areata with exclamation point hairs at the periphery of the lesions. There was no erythema or erosion associated with the lesions. The frontal lesions were skin‐colored papules that felt uneven in touch. Seborrheic dermatitis was an incidental finding during the examination with some erythema and dandruff on the scalp although the patient had not complained of this.

## 3 METHODS

**FIGURE 3:**
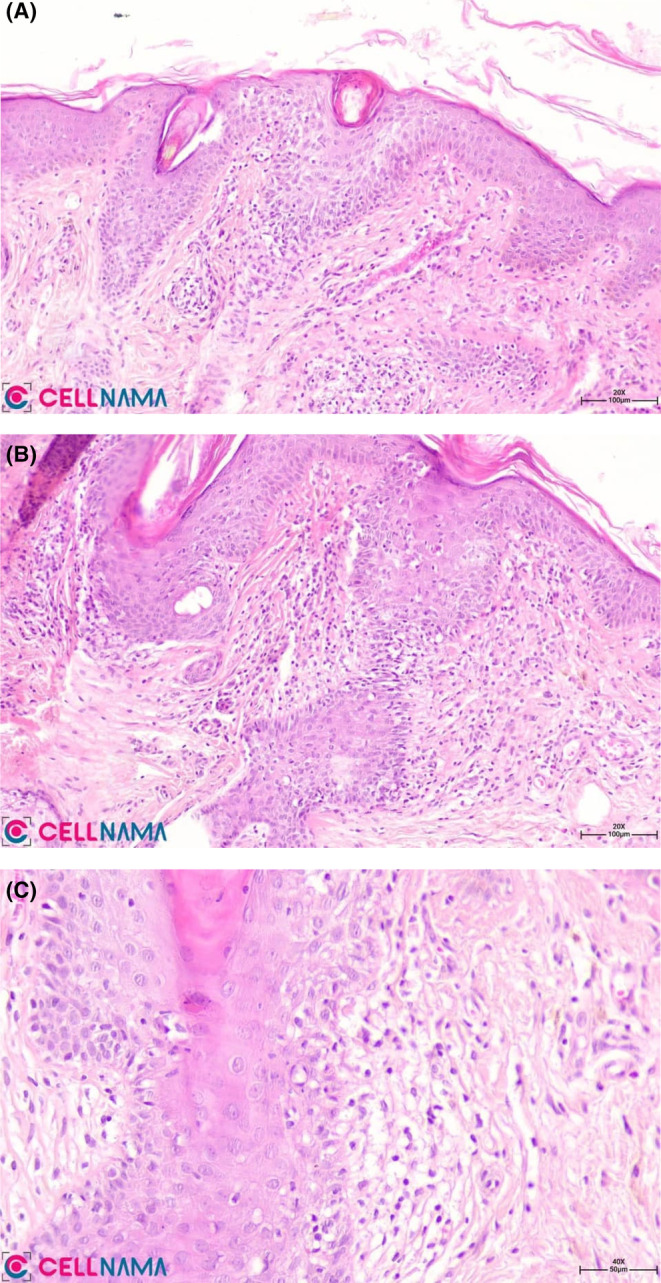
H&E: (A,B) Perifollicular lymphocytic infiltration in the superficial portions of the follicular units, ×100, ×200; (C) Perifollicular lichenoid change with basal layer vacuolar degeneration, ×400.

To confirm the diagnosis, a biopsy of the facial lesion was obtained, which was affirmative for frontal fibrosing alopecia. Histopathologic examination presented perifollicular lymphocytic infiltration, mainly around the isthmus and infundibulum portions, with basal layer vacuolar degeneration of the follicular epithelium. Villus hair follicles were also involved. A decrease in follicular density as well as a true fibrous band were observed (Figure 3). For treatment of the facial papules, isotretinoin (20 mg daily) and mometasone ointment were initiated to which the patient has responded well.

## 4 CONCLUSION AND RESULTS

The ophiasis was treated with topical minoxidil and intralesional injection of triamcinolone acetonide and responded well to the treatment. At the follow‐up visit after 8 weeks of treatment, complete symptom resolution was observed for all lesions.

Consistent with previous case reports, this paper highlights the possibility of FFA associated with AA at an especially young age. A concomitant shared pathophysiology is suspected to underlie this association and requires further investigation by researchers.

## 5 DISCUSSION

To date, cases of FFA associated with AA have been rarely reported in the literature.^10^, ^11^, ^12^ To the best of our knowledge, this is the first case of ophiasis pattern AA associated with FFA in a premenopausal female to be reported. Although FFA mainly affects postmenopausal women, cases of younger females and males have been reported.^13^ Interestingly, our patient manifested the symptoms in the early years of life, which also has been rarely reported in the literature. A summary of the reported cases is presented in Table 1.

**TABLE 1:** summary of the reported cases.

Authors	Publication date	Age	Gender	Clinical findings	Histological findings	Trichoscopy
Lin et al.^12^	2018	58	Female	Frontal hair loss with hypopigmentation, scarring, erythema, and perifollicular scale. Partial loss of eyebrows.	Lymphocyte mediated scarring, with perivascular and perifollicular lymphocytic infiltrate concentrated around the hair bulge area.	
Lin et al.^12^	2018	67	Female	Ophiasis pattern hair loss with loss of follicles, mild erythema and scale. Partial loss of eyebrows and eyelashes.	Lymphocyte mediated scarring, with perivascular and perifollicular lymphocytic infiltrate concentrated around the hair bulge area.	
Desai et al.^10^	2020	35	Female	Reduced hair density and small ill‐defined hairless patches involving frontal hairline; large hairless patches on central and parietal scalp; eyebrow loss; pull test positive for early telogen hairs	Frontal hairline Peribulbar lymphocytic infiltrate (swarm of bees); perifollicular lichenoid infiltrate and fibrosis at the isthmus	Frontal hairline with individual broken hairs and loss of vellus hairs
Desai et al.^10^	2020	65	Female	Ophiasis AA involving posterior, sides, and frontal scalp; individual terminal hairs on frontal hairline; reduced hair density on central and parietal scalp; eyebrow loss; body hair loss	Frontal scalp Perifollicular fibrosis and lichenoid infiltrate	Yellow dots in ophiasis band; peripilar casts of the lonely terminal hairs on frontal scalp
Desai et al.^10^	2020	49	Female	Irregular hair loss of frontal hairline; ill‐defined hairless patches on sideburns; decreased eyebrow density	Frontal and temporal scalp Loss of sebaceous glands; perifollicular infiltrate and perifollicular fibrosis at isthmus	Peripilar casts and loss of vellus hairs on frontotemporal scalp
Desai et al.^10^	2020	58	Female	Frontal hair loss with hypopigmentation, scarring, erythema and perifollicular scale; partial loss of eyebrows; eyelashes spared	Perivascular and perifollicular lymphocytic infiltrate at bulge; dermal fibrosis; loss of sebaceous glands and follicles	
Desai et al.^10^	2020	67	Female	Ophiasis AA; frontal hairline with loss of follicles, erythema, scale, hypopigmentation and cutaneous atrophy; partial loss of eyebrows and eyelashes	Perivascular and perifollicular lymphocytic infiltrate around bulge; perifollicular fibrosis	
Desai et al.^10^	2020	53	Female	Symmetric band‐like recession of frontotemporal hairline with hypopigmentation; well‐defined patch of hair loss on occipital scalp; normal eyebrows and eyelashes	Frontal hairline Perifollicular lymphocytic infiltrate around isthmus of hair follicles and sparing the bulb	Frontal hairline with loss of vellus hairs, loss of follicular openings, perifollicular hyperkeratosis and lone hairs; occipital scalp with broken hairs and yellow and black dots
Souissi^11^	2019	53	Female	Symmetric bandlike recession of the fronto‐temporal hairline leaving uniformly pale skin. A well circumscribed, 10 cm, patch of non‐scarring alopecia in occipital area. Absence of vellous hairs, a loss of follicular openings, a perifollicular hyperkeratosis and lone hairs in frontal area.	Prominent perifollicular lymphocytic infiltrate in a lichenoid pattern that targets the isthmus of follicles and spares the hair bulb.	

FFA and AA are diverse autoimmune variants of alopecia. Studies have suggested that the coexistence of both disorders is not incidental. Desai et al.^10^ have reported seven cases of AA associated with FFA. In their study, four out of seven manifestations and diagnoses of AA preceded the development of FFA. In the other three cases, both disorders were diagnosed at the same time and the histopathological manifestations of FFA and AA were evident in one specimen. This suggests a foregoing pathophysiology of FFA, which may provoke further development of FFA. Similar results have been reported by Lin et al.^12^ Consistent with earlier reports, our case developed ophiasis at 4 years of age and later developed FFA at 16 years of age.

Pathophysiologically, both disorders manifested with the loss of immune privilege of the hair follicles.^5^ FFA, as a variant of lymphocytic cicatricial alopecia, is characterized by irreversible scarring alopecia at the frontotemporal hairline and is associated with hair loss of the eyebrow and body.^14^, ^15^ Pathological findings include lymphocytic inflammatory infiltrate surrounding the bulge of the hair follicle and perifollicular concentric fibrosis, causing follicular damage and fibrosis.^14^

AA manifests with alopecic patches on the scalp.^14^ The main histopathological findings are lymphocytic infiltrate around the bulb of the hair follicles, an increasing number of telogen follicles and miniaturization of hair follicles.^14^

The hair follicle is considered as an immune privilege site. Because it is able to evade the immune system by downregulation of major histocompatibility complex proteins, increasing the production of local immunosuppressants and impaired antigen‐presenting cells act as an extracellular matrix barrier that inhibits disruption by immune cells. This immune privilege site was considered to be limited to the anagen bulb; however, recent studies have shown that it also affects the bulge, which protects the stem cells.^14^ As mentioned earlier, the site of the inflammatory infiltrate is a major difference between AA and FFA, which affect the bulb and bulge, respectively.^16^ Therefore, the loss of stem cells and epithelial mesenchymal transition in the bulge of follicles that have been affected by FFA can result in further scarring.^16^ However, it is as yet unclear whether or not an underlying disorder triggers the immune system to target hair follicles simultaneously in these two conditions or if they are caused by disorders of the immune system that manifest at the same time. Further research is highly encouraged in this regard.

A diagnosis can be made based on clinical findings along with histopathological studies for both conditions. Treatment commonly includes intralesional and topical steroids, minoxidil, excimer laser, finasteride, and hydroxychloroquine for FFA and corticosteroids, minoxidil, anthralin, and immunosuppressants for AA.^14^, ^17^ Our case responded well to treatment with isotretinoin with topical corticosteroid for FFA and minoxidil with intralesional corticosteroids for ophiasis. Although ophiasis is usually refractory to treatment, complete symptom resolution for all lesions was observed after 8 months in our case.

## AUTHOR CONTRIBUTIONS

**Mahsa Babaei:** Data curation; investigation; project administration; resources; supervision; writing – original draft; writing – review and editing. **Ghasem Rahmatpour Rokni:** Conceptualization; investigation; project administration; supervision; visualization. **Fatemeh Montazer:** Conceptualization; investigation; supervision; validation; visualization. **Nasim Gholizadeh:** Conceptualization; investigation; resources; visualization; writing – original draft.

## FUNDING INFORMATION

None of the authors received any funding for this submission.

## CONFLICT OF INTEREST STATEMENT

The authors have no conflict of interests for this submission.

## ETHICS STATEMENT

All content of this research adheres with the ethical guidelines developed by the Committee on Publication Ethics (COPE) during the 2nd World Conference on Research Integrity in Singapore in 2010. All parts of this study meets the Code of Conduct (the Ethics Code) and adheres to the legal requirements of the study country, Iran.

## CONSENT

Written informed consent was obtained from the patient to publish this report in accordance with the journal's patient consent policy.

## Data Availability

Data is available through contacting the corresponding author.
